# Exploring the Effects of Attribute Framing and Popularity Cueing on Hearing Aid Purchase Likelihood

**DOI:** 10.3390/audiolres16010012

**Published:** 2026-01-17

**Authors:** Craig Richard St. Jean, Jacqueline Cummine, Gurjit Singh, William (Bill) Hodgetts

**Affiliations:** 1 Department of Communication Sciences and Disorders, University of Alberta, 6-133 Clinical Sciences Building, 11304-83 Ave NW, Edmonton, AB T6G 2G3, Canada; crstjean@ualberta.ca (C.R.S.J.); jcummine@ualberta.ca (J.C.); 2 Sonova Canada, Kitchener, ON N2E 1Y6, Canada; gurjit.singh@sonova.com; 3 Department of Psychology, Toronto Metropolitan University, Toronto, ON M5B 2K3, Canada; 4 Department of Speech-Language Pathology, University of Toronto, Toronto, ON M5G 1V7, Canada

**Keywords:** message framing, purchase likelihood, social norms, teleaudiology, health psychology, hearing aids

## Abstract

**Background/Objectives:** This study explored how attribute framing (lifestyle-focused vs. technology-focused product descriptions) and popularity cueing (presence or absence of a “best-seller” label) influenced purchase likelihood for a fictitious selection of hearing aids (HAs) among Canadian adults aged 40 years and above. The study further aimed to investigate whether the effects observed were unique to HAs or applicable to less-specialized consumer technology contexts. **Method:** A 2 × 2 × 2 mixed experimental design compared attribute framing and popularity cueing effects across HAs and notebook computers at three technology levels (entry-level, midrange, and premium). Participants (*n* = 122) provided ratings indicating their purchase likelihood for each product. **Results:** Attribute framing showed no significant influence on purchase decisions across technology levels. The presence of a popularity cue that the midrange HA was the best-seller negatively affected purchase likelihood for the entry-level HA, with higher purchase likelihood ratings observed when this cue was absent. Participants expressed stronger purchase likelihood for premium HAs compared to premium notebook computers. Notably, these two effects were not statistically significant following correction for multiple comparisons. **Conclusions:** Popularity cues for HAs may have inadvertent consequences for consumer perceptions of models with differing technology levels. Findings also suggest potentially greater willingness to invest in premium health-related technologies versus familiar consumer technology. Further research involving current HA users or candidates is needed to better understand these findings.

## 1. Introduction

Hearing aid (HA) uptake, particularly among older adults with age-related hearing loss, remains persistently low, despite HAs being the most effective treatment available. Figures from *MarkeTrak 10* indicate that, among an estimated 35 million Americans who could benefit from HAs, only about 42% of individuals ages 65+ own one [1]. This gap in adoption is driven by several factors, including financial constraints, limited insurance coverage, a lack of awareness of perceived need, and stigma related to hearing aids, hearing loss, and/or ageing [2,3,4]. Prior research has primarily examined demographic and contextual predictors of HA adoption, but relatively few studies have taken an experimental approach to investigate how messaging strategies influence purchasing decisions [5]. Some researchers suggest that applying psychological theories of decision-making, such as Nudge Theory, could provide new insights into HA uptake by revealing how the framing of information influences consumer behaviour [5]. This study applies Nudge Theory to examine whether attribute framing and popularity cues can shape HA purchase decisions.

### 1.1. Nudge Theory and Decision-Making

Nudge Theory was introduced by Thaler and Sunstein [6] and has since been widely applied in behavioural economics and public policy, notably by the UK’s Behavioural Insights Team [7]. Nudging refers to subtle changes in how choices are presented to influence decision-making without restricting options [8]. Hansen and Jespersen [9] classify nudges into two categories: Type 1 nudges influence automatic, intuitive thought processes, such as placing healthy food at eye level to encourage better choices [10]. Type 2 nudges target deliberative, reflective thinking, such as persuasive messaging that portrays a decision in a particular way. This study focuses on two Type 2 nudging strategies commonly used in consumer behaviour: message framing—highlighting either the lifestyle benefits or technological capabilities of HAs, and social norm messaging—using ‘popularity cues’ (e.g., “best-selling model”) to influence consumer decisions.

### 1.2. Message Framing in Hearing Aid Marketing

Message framing refers to how information is presented to shape consumer perception. While most hearing healthcare research has focused on gain vs. loss framing (e.g., [1,2,3,4,5,6,7,8,9,10,11,12,13]), attribute framing (emphasizing product features or benefits) is more commonly used in marketing and advertising [14]. For HAs, attribute framing might emphasize technological features (e.g., advanced noise reduction, AI-driven customization) or lifestyle benefits (e.g., better social engagement, improved quality of life).

Prior research suggests that attribute framing can influence willingness to pay. For example, Amlani et al. [15] found that consumers were more willing to invest in premium HA features when descriptions emphasized patient benefits rather than technical specifications. Similarly, the way medical language is used can shape attitudes toward hearing loss and HAs. One recent study found that family physicians’ use of medical terminology could lead to more negative attitudes toward hearing loss but more positive attitudes toward HAs [16]. Conversely, another indicated that the use of medical language in newspaper articles about hearing loss might result in negative attitudes toward HA use [17]. Given the potential impact of framing on both medical and consumer decision-making, this study explores whether presenting HAs through a lifestyle vs. technology-focused lens affects purchase likelihood.

### 1.3. Social Norm Messaging and Popularity Cues

Social norm messaging refers to attempting to influence behaviour by describing what the majority of people do [18]. It can be descriptive (i.e., ‘most people choose this option’) or injunctive (i.e., ‘this is the recommended choice’). A meta-analysis of 110 studies found that descriptive norms significantly influence attitudes (*d* = 0.168) and behaviours (*d* = 0.097), while injunctive norms had a stronger effect on attitudes (*d* = 0.335) and behaviours (*d* = 0.201) [19]. A specific form of descriptive norm messaging in marketing is popularity cueing, which highlights best-selling or highly rated products [20]. Popularity cues have been shown to generally aid consumer decision-making [21], but their effect on HA purchase decisions remains unknown.

Given that HA selection involves complex trade-offs [22,23], prospective buyers may experience information overload (i.e., too much technical information to process efficiently) [24] or choice paralysis (i.e., difficulty deciding due to the sheer number of available options) [25]. In online browsing environments, where HA buyers face an even broader array of choices or information than in clinics, popularity cues may serve as a decision-making shortcut. However, their effectiveness in hearing healthcare remains unexplored, making this an important area for investigation.

### 1.4. The Present Study

Although attribute framing and popularity cueing have been widely studied in other consumer contexts, their combined effects on HA purchase decisions remain untested. This study aims to provide new insights into how online presentation strategies impact HA decision-making. Specifically, we address the following research questions: (1) Does purchase likelihood for different levels of HA technology (entry-level, midrange, premium) vary based on attribute framing (lifestyle vs. technology) and/or the presence of a popularity cue (best-seller label)? (2) Do the same framing and popularity cue effects apply to a non-medical consumer product (notebooks)? (3) Does purchase likelihood differ between hearing aids and notebooks at each technology level? Our primary interest is in HA purchase behaviour, but by including notebook computers as a comparison, we aim to determine whether the effects of these nudges are unique to hearing healthcare or generalizable to broader consumer technology purchases. Given the repeated measures design involving two technology types, we will also conduct exploratory analyses to examine whether purchase likelihood differs as a function of interactions between technology type (HA vs. notebook) and attribute framing and/or popularity cueing.

## 2. Materials and Methods

### 2.1. Participants and Study Design

The study received ethics approval from the University of Alberta’s Research Ethics Board (Pro00091783). A total of 122 Canadian adults (aged 40+ years) were recruited via Facebook advertisements and online newsletters. Participation was restricted to Canadian residents. The study employed a 2 × 2 × 2 mixed experimental design: Within-subjects factor: decision-making for two products (Hearing Aid vs. Notebook Computer). Between-subjects factors: (1) attribute framing—lifestyle benefits vs. technological capabilities; (2) popularity cueing—presence vs. absence of a best-seller label.

For the within-subjects factor, the order in which participants were presented with the notebook computer and HA decision-making scenarios was counterbalanced, such that half the participants completed the notebook scenario before completing the HA scenario and half did the opposite. For between-subjects factors, participants were randomly assigned to one of four conditions, resulting in the following sample sizes: Condition 1 (tech framing, no popularity cue): *n* = 23; Condition 2 (tech framing, popularity cue): *n* = 25; Condition 3 (lifestyle framing, no popularity cue): *n* = 38; and Condition 4 (lifestyle framing, popularity cue): *n* = 36. Each participant experienced the same conditions for both the HA and notebook scenarios.

### 2.2. Procedure and Experimental Manipulation

#### 2.2.1. Study Recruitment and Platform

Prospective participants were invited to an online study on technology purchasing decisions. The recruitment message stated: “We are continuously presented with new types of technology we may wish to purchase. Our research team is interested in learning more about the types of technology that are of interest to consumers and the types of options they might choose. We are only interested in your opinions. This study is not an advertisement for any particular product or company.” Clicking the participation link directed users to the REDCap survey platform, where they read an information letter detailing the study tasks, provided informed consent, and completed demographic questions on age, gender, language, education, household income, employment status, and marital status.

#### 2.2.2. Decision-Making Scenarios

Participants completed two product decision-making scenarios. In the HA scenario, participants were told: “You have been diagnosed with hearing loss and have learned that you could benefit from hearing aids. You visit a hearing clinic to discuss options with an audiologist. At the clinic, you receive the following information.” They were then shown a comparison table with three fictitious HA models (QX50, QX70, QX90), each with technical specifications, descriptions, and pricing. In the notebook computer scenario, participants were told: “You are considering purchasing a new notebook computer. You look up a well-known computer manufacturer and learn that they are offering a new line of notebooks. The manufacturer describes the product options as follows.” They were then presented with a comparison table of three fictitious notebook models (z5, z7, z9) with specifications, descriptions, and pricing.

#### 2.2.3. Experimental Manipulations

Our first experimental manipulation was attribute framing (lifestyle vs. technology-focused). Our lifestyle-framed descriptions emphasized everyday benefits and user experience (e.g., “The QX70 is designed with your connected life in mind” or “You’ve never seen portability this powerful!”). See Table 1 and Table 2 for lifestyle-framed descriptions of HAs and notebooks, respectively:

Our technology-framed descriptions focused on technical features and device specifications (e.g., “The QX50 will help boost the signal the ear receives” or “the z9 additionally offers compatibility with Wifi 6 and 5 G mobile broadband”). See Table 3 and Table 4 for tech-framed descriptions of HAs and notebooks, respectively:

HA model descriptions varied from 46 to 65 words, while notebook model descriptions ranged from 38 to 53 words.

Our second experimental manipulation was either the presence or absence of a popularity cueing statement for the midrange option in the HA and notebook model descriptions. Participants in the cue-present condition saw a bolded, red-bordered statement beneath the table reading (HA Scenario) “Note: A popular tech review website states that the QX70 is the best-selling model.”; (Notebook Scenario): “Note: A popular tech review website states that the z7 is the best-selling model.” This manipulation did not specify whether the model was a best-seller among all devices or just among the three presented.

### 2.3. Experimental Measures

#### 2.3.1. Purchase Likelihood Ratings

Below each HA and notebook comparison table, participants rated their likelihood of purchasing each model using a visual analogue scale (0–100): Anchors: “Very unlikely” (0) to “Very likely” (100) slider with real-time numerical display to indicate selection. To simulate real-world decision-making, participants also rated: “I would not purchase any of the above models.” Scale: “Strongly disagree” (0) to “Strongly agree” (100). This accounted for indecision or refusal to choose [26]. Participants were not forced to allocate 100 points across options (e.g., they could rate all options highly or none at all).

#### 2.3.2. Additional Opinion Measures

Following the purchase scenarios, participants rated seven additional statements using visual analogue scales (0–100): (1) “I consider myself knowledgeable about notebook computers”; (2) “Notebook computers are important to me”; (3) “I consider myself knowledgeable about hearing aids”; (4) “Hearing aids are important to me”; (5) “I think my hearing in general is…”; (6) “I think my hearing in quiet is…”; (7) “I think my hearing in background noise is…”. The anchors for statements 1–4 were Strongly Disagree (0) to Strongly Agree (100), while the anchors for statements 5–7 were Bad (0) to Excellent (100). These measures were included because prior research suggests knowledge and personal relevance may moderate framing effects [27,28] and severity of hearing loss predicts willingness to adopt HAs [29]. These responses were used as covariates in subsequent analyses. Finally, participants were asked: “Do you, or have you ever, worn hearing aids?” (Yes/No).

## 3. Results

One hundred and twenty-two participants completed the study. We inspected the data for outliers using a z-score transformation for each of our outcome measures. Data points with standardized values equal to or greater than +/−2.0 [30] were found for the z7 and “Would not purchase…” outcomes in the notebook scenario and for the “Would not purchase…” outcome in the HA scenario, and were removed from the dataset. In total, 20 data points out of 976, or 2.05% of the total data points across all dependent variables, were removed.

### 3.1. Demographic Information

Of the 122 total participants, 96 identified as female (78.7%), 25 identified as male (20.5%), and 1 identified as ‘Other’ (0.82%). One hundred and eighteen reported English as their primary language spoken at home (96.7%), while one reported French, and three reported ‘Other’. Fifty-one (41.8%) reported a Master’s degree or higher as their highest level of education completed, while forty-five (36.9%) reported a Bachelor’s degree (24.4%). Seventy-nine reported their employment status as employed full time (65.1%), while eighty-six reported (70.5%) reported their marital status as married/common law (76.7%). More than half reported (54.2%) a household income of over CAD 100,000, with CAD 75,000–CAD 99,999 (22.1%) as the second most frequent income category. One hundred and sixteen (95.1%) participants stated that they were not currently using, nor had they ever used, HAs to treat hearing loss; the other six participants (4.9%) indicated that they were or had. The mean age of the sample was 50.9 (*SD* = 8.47) years. A full breakdown of demographic categories is displayed in Table 5.

### 3.2. Main and Interaction Effects of Framing and Popularity Cueing

For our primary analysis, we conducted four 2 (attribute framing: lifestyle vs. technology) × 2 (popularity cue: present vs. absent) × 2 (technology type: HA vs. notebook computer) mixed analyses of covariance (ANCOVA). Our between-subjects factors (attribute framing, popularity cueing) compared the effects of lifestyle vs. tech framing and presence vs. absence of a popularity cue on participants’ self-rated likelihood of purchasing the QX50, QX70, and QX90 HAs, and the z5, z7, and z9 notebooks, or none of the above for each technology type. Our within-subjects factor (technology type) compared whether purchase likelihood for each level of technology (entry-level: QX50 vs. z5; midrange: QX70 vs. z7; premium: QX90 vs. z9, or none of the above in each case) differed depending on whether participants were deciding on HAs vs. notebook computers. This analytical approach also allowed us to examine interaction effects between the within-subjects factor and both between-subjects factors. Our seven covariates were participants’ subjective ratings of knowledge about HAs, subjective ratings of knowledge about notebook computers, the importance HAs held for them, the importance notebook computers held for them, and their ability to hear in general, in quiet, and in background noise. Across our four main analyses of covariance, we applied a Bonferroni-corrected alpha level (adjusted α = 0.05/4 = 0.0125) to mitigate the inflated Type I error rate. We present our full results below. See Table 6 for a full breakdown of means of purchase likelihood for HA and notebook models by experimental condition.

#### 3.2.1. Entry-Level (QX50 HA, z5 Notebook)

**Figure 1:**
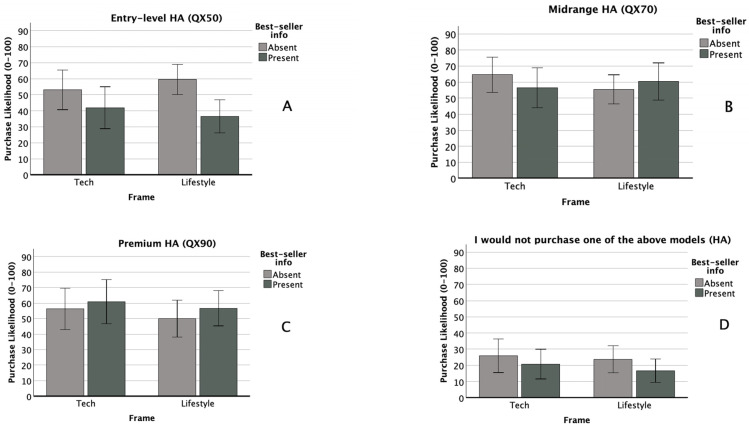
HA purchase likelihood mean comparisons by between-subjects factors (frame type, popularity cueing).

**Figure 2:**
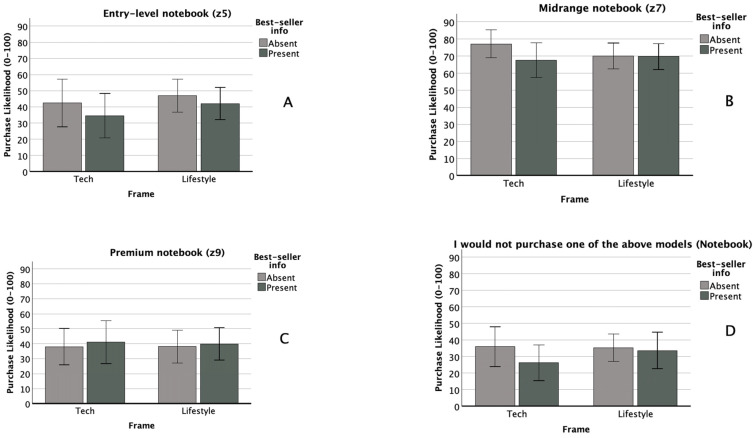
Notebook purchase likelihood mean comparisons by between-subjects factors (frame type, popularity cueing).

**Figure 3:**
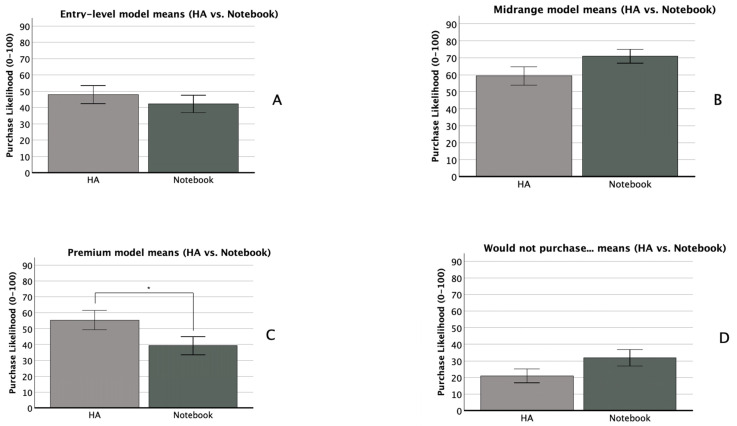
Purchase likelihood at each technology level by device type (HA vs. notebook computer). * indicates significant difference at *p* < 0.001.

There was no significant within-subjects effect of technology type (*F*(1, 111) = 0.09, *p* = 0.764, *η_p_*^2^ = 0.000), nor were any within- × between-subjects interactions significant. Observed effect sizes for the technology type × attribute framing, technology type × popularity cueing, and three-way interactions were *η_p_*^2^ = 0.009, 0.021, and 0.010, respectively. We observed a moderate between-subjects main effect of popularity cueing (*F*(1, 111) = 6.262, *p* = 0.014, *η_p_*^2^ = 0.053), with a *p* value just above our adjusted alpha level of 0.0125. Post hoc testing showed that this effect was driven by a significant difference in the HA scenario, where purchase likelihood was greater when the popularity cue for the mid-level technology was absent (*M* = 57.1, *SD* = 28.5) than when it was present (*M* = 38.7, *SD* = 30.7), *t*(120) = 3.42, *p* = <0.001, *d* = 0.620. There was no significant between-subjects main effect of attribute framing (*F*(1, 111) = 2.322, *p* = 0.130, *η_p_*^2^ = 0.020), nor was the between-subjects interaction significant (*F*(1, 111) = 0.559, *p* = 0.456, *η_p_*^2^ = 0.005). Self-rated notebook knowledge was a significant covariate (*F*(1, 111) = 7.166, *p* = 0.009, *η_p_*^2^ = 0.061). Figure 1A and Figure 2A compare purchase likelihood means by between-subjects factors for the entry-level HA and notebook conditions, respectively. Figure 3A compares entry-level purchase likelihood means by technology type, the within-subjects factor.

#### 3.2.2. Midrange (QX70 HA, z7 Notebook)

There was no significant within-subjects effect of technology type (*F*(1, 102) = 1.311, *p* = 0.255, *η_p_*^2^ = 0.013), nor were any within- × between-subjects interactions significant. Observed effect sizes for the technology type × attribute framing, technology type × popularity cueing, and three-way interactions were *η_p_*^2^ = 0.003, 0.013, and 0.002, respectively. There were no significant between-subjects main effects of attribute framing (*F*(1, 102) = 0.979, *p* = 0.325, *η_p_*^2^ = 0.010) or popularity cueing (*F*(1, 102) = 0.469, *p* = 0.495, *η_p_*^2^ = 0.005). The between-subjects interaction was likewise non-significant (*F*(1, 102) = 2.722, *p* = 0.102, *η_p_*^2^ = 0.026). No covariates were significant. Figure 1B and Figure 2B compare purchase likelihood means by between-subjects factors for the midrange HA and notebook conditions, respectively. Figure 3B compares midrange purchase likelihood means by technology type, the within-subjects factor.

#### 3.2.3. Premium (QX90 HA, z9 Notebook)

There was a small within-subjects effect of technology type (*F*(1, 111) = 4.233, *p* = 0.042, *η_p_*^2^ = 0.037), although the *p* value exceeded our adjusted alpha level of 0.0125. A paired samples *t*-test indicated that participants reported higher purchase likelihood for the premium HA model (*M* = 55.4, *SD* = 34.0) than for the premium notebook model (*M* = 39.2, *SD* = 32.0), *t*(122) = 4.61, *p* = <0.001, and *d* = 0.42 (see Figure 3C). No within- × between-subjects factor interactions were significant. Observed effect sizes for the technology type × attribute framing, technology type × popularity cueing, and three-way interactions were *η_p_*^2^ = 0.000, 0.001, and 0.000, respectively. There were no significant between-subjects main effects of attribute framing (*F*(1, 111) = 0.708, *p* = 0.402, *η_p_*^2^ = 0.006) or popularity cueing (*F*(1, 111) = 0.542, *p* = 0.463, *η_p_*^2^ = 0.005). The between-subjects interaction was likewise non-significant (*F*(1, 111) = 0.082, *p* = 0.775, *η_p_*^2^ = 0.000). No covariates were significant. Figure 1C and Figure 2C compare purchase likelihood means by between-subjects factors for the midrange HA and notebook, respectively. Figure 3C compares premium purchase likelihood means by technology type, our within-subjects factor.

#### 3.2.4. I Would Not Purchase Any of the Above Models (HA and Notebook)

There were no significant within-subjects effects of technology type (*F*(1, 105) = 0.364, *p* = 0.548, *η_p_*^2^ = 0.003), nor were any within- × between-subjects interactions significant. Observed effect sizes for the technology type × attribute framing, technology type × popularity cueing, and three-way interactions were *η_p_*^2^ = 0.007, 0.000, and 0.001, respectively. HA importance was a significant covariate in the within-subjects model (*F*(1, 105) = 4.070, *p* = 0.046, *η_p_*^2^ = 0.037). There were no significant between-subjects main effects of attribute framing (*F*(1, 105) = 0.000, *p* = 0.985, *η_p_*^2^ = 0.000) or popularity cueing (*F*(1, 105) = 0.560, *p* = 0.456, *η_p_*^2^ = 0.005). The between-subjects interaction was likewise non-significant (*F*(1, 105) = 0.114, *p* = 0.736, *η_p_*^2^ = 0.001). No covariates were significant in the between-subjects model. Figure 1D and Figure 2D compare purchase likelihood means by between-subjects factors for the midrange HA and notebook, respectively. Figure 3D compares the likelihood of non-purchase means by technology type, our within-subjects factor.

## 4. Discussion

This study examined whether self-reported HA purchase likelihood was influenced by two factors: (1) attribute framing—whether descriptions emphasized lifestyle benefits or technological features, and (2) popularity cueing—whether the midrange model was labelled as a best-seller. Covariates included HA and notebook knowledge, product importance, and perceived hearing ability in different environments. These variables were selected due to their potential relevance in technology decision-making. Popularity cueing negatively affected the purchase likelihood of the entry-level HA (*η_p_*^2^ = 0.053): participants rated it lower when a popularity cue was present. Premium HAs were preferred over premium notebooks (*η_p_*^2^ = 0.037). However, these effects did not reach statistical significance after Bonferroni correction (*α* = 0.0125), highlighting the need for cautious interpretation.

### 4.1. Effect of Popularity Cueing on HA Purchase Likelihood

A subset of participants was presented with a best-seller label for the midrange HA, while others did not. Typically, popularity cues simplify decision-making and increase product appeal [21]. However, in this study, popularity cueing did not exert a substantively meaningful influence on purchase likelihood for the midrange HA, whereas it produced a moderately sized negative effect for the entry-level HA.

#### 4.1.1. Why Did Popularity Cueing Reduce Interest in the Entry-Level HA?

The best-seller label may have devalued the entry-level option, making it seem less desirable in comparison. Without the cue, participants may have perceived the entry-level model as a good value, whereas the cue may have reframed it as inferior or ‘cheap.’

#### 4.1.2. Why Did the Popularity Cue Not Increase Midrange HA Purchase Likelihood?

Baseline preference may have already been high: the midrange HA had the highest purchase likelihood rating (*M* = 58.84), exceeding both the premium (*M* = 55.38) and entry-level (*M* = 47.92). The presentation of three differently priced models may have invoked a compromise effect [31]: participants may have naturally gravitated toward the middle option as a ‘safe’ choice regardless of the popularity cue. Mean purchase likelihood for the middle option was highest for both the HA and notebook scenarios, which lends support to this interpretation.

#### 4.1.3. Why Did the Popularity Cue Not Affect the Premium HA?

Higher-income participants (64.8% full-time employed, 78.7% university graduates, 67% with a household income over CAD 100,000) may have seen premium HAs as a status purchase, unaffected by social proof. Further, unlike the entry-level model, the premium HA may not have suffered by comparison, since premium buyers may not base decisions on majority preferences.

### 4.2. Effect of Attribute Framing on HA Purchase Likelihood

#### Why Was There No Meaningful Effect of Lifestyle vs. Technology Framing?

There may have been minimal differences between the attribute frames: both descriptions highlighted the same features, even if phrased differently. Participants may have focused on feature lists and pricing: eye-tracking studies suggest consumers often skim for key specs rather than engaging deeply with descriptions [32]. Additionally, framing may not influence HA buyers strongly: real-world HA advertisements often combine both lifestyle and technical descriptions, reducing the impact of artificial framing manipulations.

### 4.3. HAs vs. Notebook Computers

While our primary interest was the potential influence of our experimental manipulations on HA purchase likelihood, we also wondered about the extent to which purchase likelihood would differ between HAs and notebooks at each level of technology. In terms of statistical significance, we only observed an initially significant within-subjects effect (*p* = 0.042, *η_p_*^2^ = 0.037) in the premium level comparison (QX90 vs. z9). However, this difference was no longer statistically significant after applying our adjusted alpha value. An exploratory *t*-test to better understand the effect showed that participants indicated higher purchase likelihood for the premium HA model (*M* = 55.4, *SD* = 34.0) than for the premium notebook model (*M* = 39.2, *SD* = 32.0), *t*(122) = 4.61, *p* = <0.001, *d* = 0.42. Participants’ self-ratings of HA knowledge (*M* = 28.3, *SD* = 26.7) and notebook computer knowledge (*M* = 57.6, *SD* = 22.8) may provide a potential explanation. With perceived notebook knowledge twice as high as perceived HA knowledge, it may be assumed that our sample has a clearer idea of what features they require in a notebook, a common piece of technology, than in a HA, a specialty piece of technology. This difference points to the possibility that consumers may generally be more willing to pay for premium features in health technology than in leisure or business technology, possibly due to lesser knowledge about whether premium features are useful or essential for them. In other words, their higher willingness to pay may stem from a fear of missing out on potential benefits.

Future research may wish to explore this further and may wish to consider whether prospect theory, particularly loss aversion [33], provides useful insight into the question. An additional and possibly related factor for future research to consider is whether such motivations may stem from an increased concern about making the wrong choice, given that health technology decisions may register as having higher stakes than decisions on more common technological products.

### 4.4. Limitations

Some potential limitations of the study are as follows. First, some of the characteristics of the sample may reduce the generalizability of our findings. In particular, the sample was predominantly female (79%), highly educated (78% with a bachelor’s degree or higher), and of higher income (55% earning a household income of over CAD 100 K+). These characteristics diverge from the Canadian demographic patterns of hearing loss and hearing aid candidacy. Population-level audiometric data indicate hearing loss is more frequent among men than women, among those without post-secondary education than those with post-secondary education, and among members of households earning under CAD 100 K than those earning over CAD 100 K [34]. Regarding income, Statistics Canada reports that among those who could benefit from a HA but do not have one, 72% cite cost as a barrier [35]. With higher earners overrepresented in our sample, the purchase likelihood ratings reported here may not be generalizable at the population level.

Second, participants were predominantly not HA seekers. The study asked participants to imagine having hearing loss, which may not fully capture the emotional and financial considerations of real HA buyers. Third, the decision-making context was artificial. Unlike real-world HA purchases, where peer reviews, audiologist recommendations, and trials play a role, this study only presented textual descriptions.

Fourth, the decision-making scenarios in this study may not generalize to markets outside Canada. In the United States, recent legislative reclassification of HAs to include over-the-counter (OTC) devices and headphones with hearing-assistive features has introduced a new set of more easily accessible and lower-cost options intended for individuals with mild-to-moderate hearing loss [35,36]. The *MarkeTrak 2025* survey indicates that OTC devices have increased in popularity, particularly among younger, non-white, and cost-sensitive populations [35]. OTC buyers also tend to be more brand-conscious than prescription HA buyers [37], and tend to value social media-based reviews and recommendations to guide their decision-making [35]. The nature of the prescription devices and the “best-seller” popularity cue used in our study, in addition to our sample characteristics and Canadian recruitment, may limit the relevance of our findings with respect to informing marketing strategies for non-prescription HAs.

## 5. Conclusions

This study provides insight into how popularity cues and attribute framing may influence HA purchase decisions. While midrange popularity cueing had a moderately sized negative effect on entry-level HA appeal, it had a negligibly sized effect on midrange HA appeal itself. Additionally, attribute framing had an overall negligible effect on purchase likelihood, suggesting that HA descriptions must differ more substantially to create an impact. A further finding was a small but statistically non-significant effect for technology type, in which participants demonstrated greater purchase likelihood for premium HAs than for premium notebook computers. These findings contribute to a deeper understanding of HA marketing strategies and provide a foundation for future work on decision-making in assistive technology purchases. Future research should target actual HA buyers rather than general consumers, investigate real-world HA marketing through qualitative methods and eye-tracking studies, and further explore factors surrounding willingness to pay for premium HA features.

In summary, when it comes to HA purchases, nudges like popularity cues and lifestyle framing may not land the way we expect. As shown here, ‘social proof’ (such as advertising a HA model as the best-selling option) may do more harm than good for lower-priced models. These results highlight the need for hearing care providers and marketers to think carefully about how they present options, especially in emotionally and financially complex decision-making contexts. In short, nudges need nuance: what works for selling familiar technology like notebook computers may not necessarily work for hearing aids.

## Figures and Tables

**Table 1:** Hearing aid descriptions used in the lifestyle-focused condition.

Model	Description	Features	Price
QX50	The QX50 will help you hear better and will allow you to more easily enjoy sound in your daily life. For instance, the radio in your car and conversations with friends will sound louder and crisper. Feedback cancellation minimizes distracting noises, allowing you to focus on the sounds you want to hear.	Directional microphone Feedback cancellation	CAD 1900
QX70	The QX70 is designed with your connected life in mind. Smart device streaming lets you answer phone calls and enjoy the sound from your favorite TV shows directly through your hearing aid. The QX70 is also adjustable via an app, allowing you to customize your listening settings to suit your preferences.	Directional microphone Feedback cancellation Smart device streaming Adjustable via app	CAD 2600
QX90	The QX90 is the most advanced device, and will help you hear your best in the widest range of today’s situations. The Mask Mode and StereoZoom features are designed with noisy and physically-distanced environments in mind, allowing you to continue hearing as clearly as possible in challenging situations. These features also help reduce the effort it takes when you’re trying to listen in these environments.	Directional microphone Feedback cancellation Smart device streaming Adjustable via app Mask Mode StereoZoom	CAD 3300

**Table 2:** Notebook descriptions used in the lifestyle-focused condition.

Model	Description	Features	Price
z5	Whether you’re zipping across town or Zooming from your bedroom, the z5 offers maximum portability (weighing in at just 900 g!) without sacrificing the performance you require. Built for entertainment and basic work/study needs, this unit is ideal for staying connected on the go.	11-inch display 4-core z-processor 16 GB memory 2 TB storage Up to 12 h battery life Backlit Keyboard	CAD 1399
z7	The z7 strikes a sweet spot for work, play, and everything in between. At an even 1000 g, this 13-inch unit is ideal at your work desk, kitchen table, or your favorite comfy spot in the living room. Its Touch ID feature makes privacy faster and easier than ever.	13-inch display 6-core z-processor 32 GB memory 4 TB storage Up to 16 h battery life Touch Bar and Touch ID Backlit keyboard	CAD 1699
z9	You’ve never seen portability this powerful! The z9 packs features for every lifestyle into a unit the weight of your water bottle. Its full-day’s worth of battery life ensures you’ll never be caught without a charge. With top-of-the-line connectivity, processing, and security features, the z9 is the ultimate notebook for your connected life.	15-inch Retina display 8-core z-processor 64 GB memory 8 TB storage Up to 24 h battery life Supports Wifi 6 Supports 5 G mobile Touch Bar and Touch ID Backlit keyboard	CAD 2199

**Table 3:** Hearing aid descriptions used in the technology-focused condition.

Model	Description	Features	Price
QX50	The QX50 will help boost the signal the ear receives. The key feature is a directional microphone, which makes the target signal louder than the background noise. This device also comes with feedback cancellation to minimize squeaking sounds from the device that sometimes happen with hearing aids.	Directional microphone Feedback cancellation	CAD 1900
QX70	The QX70 includes all the features of the QX50 and offers the extra benefit of audio streaming with smart devices, like phones or TVs. Additionally, the QX70 is compatible with an iPhone/Android app that allows the device to be adjusted comfortably in various listening settings.	Directional microphone Feedback cancellation Smart device streaming Adjustable via app	CAD 2600
QX90	The QX90 offers the most advanced functionality. Including all the features of the QX70, this model also introduces Mask Mode and StereoZoom. Mask Mode uses artificial intelligence to boost speech clarity in difficult listening situations. StereoZoom makes it easier for the ear to differentiate between speech coming from multiple directions by focusing on target sounds.	Directional microphone Feedback cancellation Smart device streaming Adjustable via app Mask Mode StereoZoom	CAD 3300

**Table 4:** Notebook descriptions used in the technology-focused condition.

Model	Description	Features	Price
z5	At only 900 g, the z5 offers impressive processing power and battery life for its small size. Its 4-core processor outperforms comparably-sized models on the market, while its 2 TBs of storage far surpasses the storage capacity of other mini-notebooks.	11-inch display 4-core z-processor 16 GB memory 2 TB storage Up to 12 h battery life Backlit Keyboard	CAD 1399
z7	The standard-sized z7 maintains a light weight and ease of portability while presenting a significant upgrade in all other major features: longer battery life, faster processing, and increased storage. It also comes equipped with a Touch Bar and Touch ID, features which represent market leading advancements in accessibility and privacy.	13-inch display 6-core z-processor 32 GB memory 4 TB storage Up to 16 h battery life Touch Bar and Touch ID Backlit keyboard	CAD 1699
z9	With a 15-inch Retina display, this is the largest model in the z-line, and by far the most powerful. With double the capacity and performance of the z7, the z9’s additionally offers compatibility with Wifi 6 and 5G mobile broadband, altogether representing the cutting edge in notebook computing.	15-inch Retina display 8-core z-processor 64 GB memory 8 TB storage Up to 24 h battery life Supports Wifi 6 Supports 5 G mobile Touch Bar and Touch ID Backlit keyboard	CAD 2199

**Table 5:** Demographic category frequency counts.

Characteristic	Category	*N*	Percentage
Highest level of education completed	High school	1	0.8
	Trade/technical/vocational	7	5.7
	Some university/college	18	14.7
	Bachelor’s degree	45	36.9
	Master’s degree or higher	51	41.8
Employment status	Employed part-time	11	9.0
	Employed full-time	79	64.8
	Retired	10	8.2
	Student	16	13.1
	Unable to work	3	2.5
	Unemployed	2	1.6
	Other	1	0.8
Marital status	Single	18	14.8
	Married (or Common Law)	86	70.5
	Separated	5	4.1
	Divorced	13	10.7
Household income (in Canadian dollars)	Under CAD 25,000	7	5.7
	CAD 25,000–CAD 39,999	5	4.1
	CAD 40,000–CAD 49,999	2	1.6
	CAD 50,000–CAD 74,999	14	11.5
	CAD 75,000–CAD 99,999	27	22.1
	Over CAD 100,000	67	54.9
Currently using or ever used HAs	No	116	95.1
	Yes	6	4.9

**Table 6:** Mean purchase likelihood for each HA and notebook model by experimental condition.

HA Model	Condition	*N*	Mean	SD
QX50	Tech/No cue	23	53.04	28.51
	Tech/Cue	25	41.92	31.58
	Lifestyle/No cue	38	59.55	28.63
	Lifestyle/Cue	36	36.33	30.26
QX70	Tech/No cue	22	64.50	26.12
	Tech/Cue	22	57.09	31.38
	Lifestyle/No cue	35	55.54	27.67
	Lifestyle/Cue	34	61.18	33.23
QX90	Tech/No cue	23	56.22	30.83
	Tech/Cue	25	60.96	34.42
	Lifestyle/No cue	38	49.97	36.36
	Lifestyle/Cue	36	56.67	33.61
Would not purchase	Tech/No cue	22	28.46	26.98
	Tech/Cue	22	21.36	21.99
	Lifestyle/No cue	36	25.00	26.60
	Lifestyle/Cue	36	20.47	25.32
**Notebook model**	**Condition**	***N***	**Mean**	**SD**
z5	Tech/No cue	23	42.39	34.09
	Tech/Cue	25	34.56	33.32
	Lifestyle/No cue	38	47.00	31.32
	Lifestyle/Cue	36	42.11	29.43
z7	Tech/No cue	22	77.18	18.43
	Tech/Cue	22	67.64	22.85
	Lifestyle/No cue	35	70.06	22.15
	Lifestyle/Cue	34	69.74	21.66
z9	Tech/No cue	23	38.04	28.18
	Tech/Cue	25	41.08	34.81
	Lifestyle/No cue	38	38.08	33.47
	Lifestyle/Cue	36	39.89	32.10
Would not purchase	Tech/No cue	22	36.00	27.08
	Tech/Cue	22	26.32	24.43
	Lifestyle/No cue	36	35.31	24.44
	Lifestyle/Cue	36	33.72	32.42

## Data Availability

The dataset collected and analyzed in this study will be made available upon reasonable request.

## Abbreviations

The following abbreviations are used in this manuscript:

HA	Hearing Aids
OTC	Over the counter

## References

[1] Jorgensen L., Novak M. (2020). Factors influencing hearing aid adoption. Semin. Hear..

[2] Ritter C.R., Barker B.A., Scharp K.M. (2020). Using attribution theory to explore the reasons adults with hearing loss do not use their hearing aids. PLoS ONE.

[3] Yong M., Willink A., McMahon C., McPherson B., Nieman C.L., Reed N.S., Lin F.R. (2019). Access to adults’ hearing aids: Policies and technologies used in eight countries. Bull. World Health Organ..

[4] Ostevik A.V., Westover L., Gynane H., Herst J., Cummine J., Hodgetts W.E. (2019). Comparison of health insurance coverage for hearing aids and other services in Alberta. Healthc. Policy.

[5] Iankilevitch M., Singh G., Russo F.A. (2023). A scoping review and field guide of theoretical approaches and recommendations to studying the decision to adopt hearing aids. Ear Hear..

[6] Thaler R.H., Sunstein C.R. (2008). Nudge: Improving Decisions About Health, Wealth, and Happiness.

[7] Junghans A.F., Cheung T.T., De Ridder D.D. (2015). Under consumers’ scrutiny: An investigation into consumers’ attitudes and concerns about nudging in the realm of health behavior. BMC Public Health.

[8] Raban M.Z., Gonzalez G., Nguyen A.D., Newell B.R., Li L., Seaman K.L., Westbrook J.I. (2023). Nudge interventions to reduce unnecessary antibiotic prescribing in primary care: A systematic review. BMJ Open.

[9] Hansen P.G., Jespersen A.M. (2013). Nudge and the manipulation of choice: A framework for the responsible use of the nudge approach to behaviour change in public policy. Eur. J. Risk Regul..

[10] Kroese F.M., Marchiori D.R., De Ridder D.T. (2016). Nudging healthy food choices: A field experiment at the train station. J. Public Health.

[11] Jean C.R.S., Cummine J., Singh G., Hodgetts W.E. (2021). Be part of the conversation: Audiology messaging during a hearing screening. Ear Hear..

[12] Hodgetts B., Ostevik A., Aalto D., Cummine J. (2017). Don’t fade into the background: A randomized trial exploring the effects of message framing in audiology. Can. J. Speech Lang. Pathol. Audiol..

[13] de Bruijn G.J., Spaans P., Jansen B., van’t Riet J. (2016). Testing the effects of a message framing intervention on intentions towards hearing loss prevention in adolescents. Health Educ. Res..

[14] Scheufele D.A., Iyengar S., Kenski K., Jamieson K.H. (2014). The state of framing research: A call for new directions. Oxford Handbook of Political Communication Theories.

[15] Amlani A.M., Taylor B., Weinberg T. (2011). Increasing hearing aid adoption rates through value-based advertising and price unbundling. Hear. Rev..

[16] Adorni R., Manzi C., Crapolicchio E., Steca P. (2022). The role of the family doctor’s language in modulating people’s attitudes towards hearing loss and hearing aids. Health Soc. Care Community.

[17] Manzi C., Adorni R., Di Cicco G., Milano V., Manunta E., Montermini F., Steca P. (2022). Implicit and explicit attitudes toward hearing aids: The role of media language. J. Lang. Soc. Psychol..

[18] Cialdini R.B., Reno R.R., Kallgren C.A. (1990). A focus theory of normative conduct: Recycling the concept of norms to reduce littering in public places. J. Pers. Soc. Psychol..

[19] Rhodes N., Shulman H.C., McClaran N. (2020). Changing norms: A meta-analytic integration of research on social norms appeals. Hum. Commun. Res..

[20] Barnes A.J., Shavitt S. (2024). Top rated or best seller? Cultural differences in responses to attitudinal versus behavioral consensus cues. J. Consum. Res..

[21] Ghiassaleh A., Kocher B., Czellar S. (2020). Best seller!? Unintended negative consequences of popularity signs on consumer choice behavior. Int. J. Res. Mark..

[22] Manchaiah V., Portnuff C., Sharma A., Swanepoel D.W. (2023). Over-the-counter hearing aids: What do consumers need to know?. Hear. J..

[23] Plyler P.N., Hausladen J., Capps M., Cox M.A. (2021). Effect of hearing aid technology level and individual characteristics on listener outcome measures. J. Speech Lang. Hear. Res..

[24] Amlani A.M. (2016). Application of the consumer decision-making model to hearing aid adoption in first-time users. Semin. Hear..

[25] Schwartz B. (2004). The Paradox of Choice: Why More Is Less.

[26] Dhar R., Simonson I. (2003). The effect of forced choice on choice. J. Mark. Res..

[27] Bullock J.B., Vedlitz A. (2017). Emphasis framing and the role of perceived knowledge: A survey experiment. Rev. Policy Res..

[28] Wirtz J.G., Sar S., Ghuge S. (2015). The moderating role of mood and personal relevance on persuasive effects of gain- and loss-framed health messages. Health Mark. Q..

[29] Meyer C., Hickson L. (2012). What factors influence help-seeking for hearing impairment and hearing aid adoption in older adults?. Int. J. Audiol..

[30] Meade A.W., Craig S.B. (2012). Identifying careless responses in survey data. Psychol. Methods.

[31] Simonson I. (1989). Choice based on reasons: The case of attraction and compromise effects. J. Consum. Res..

[32] Jin J., Wang A., Wang C., Ma Q. (2023). How do consumers perceive and process online overall vs. individual text-based reviews? Behavioral and eye-tracking evidence. Inf. Manag..

[33] Kahneman D., Tversky A. (1979). Prospect theory: An analysis of decision under risk. Econometrica.

[34] Feder K., Michaud D., Ramage-Morin P., McNamee J., Beauregard Y. (2015). Prevalence of hearing loss among Canadians aged 20 to 79: Audiometric results from the 2012/2013 Canadian Health Measures Survey. Health Rep..

[35] Jilla A.M., Jorgsensen L. (2025). Hearing aid adoption in the OTC hearing aid era: Market trends and consumer insight from MarkeTrak 2025. Semin. Hear..

[36] Picou E.M., Huang H. (2025). Are Over-the-Counter hearing aids affordable, accessible, and satisfactory? Insights from MarkeTrak 25 survey data. Semin. Hear..

[37] Dobyan B., Kihm J. (2025). MarkeTrak 2025: Consumer perspectives on hearing health in an evolving market. Semin. Hear..

